# Transcriptome sequencing of gingival biopsies from chronic periodontitis patients reveals novel gene expression and splicing patterns

**DOI:** 10.1186/s40246-016-0084-0

**Published:** 2016-08-17

**Authors:** Yong-Gun Kim, Minjung Kim, Ji Hyun Kang, Hyo Jeong Kim, Jin-Woo Park, Jae-Mok Lee, Jo-Young Suh, Jae-Young Kim, Jae-Hyung Lee, Youngkyun Lee

**Affiliations:** 1 Department of Periodontology, School of Dentistry, Kyungpook National University, Daegu, 41940 Korea; 2 Institute for Hard Tissue and Bone Regeneration, Kyungpook National University, Daegu, 41940 Korea; 3 Department of Life and Nanopharmaceutical Sciences, Kyung Hee University, Seoul, 02447 Korea; 4 Department of Biochemistry, School of Dentistry, Kyungpook National University, 2177 Dalgubeol-daero, Joong-gu, Daegu, 41940 Korea; 5 Department of Maxillofacial Biomedical Engineering, School of Dentistry, Kyung Hee University, 26 Kyunghee-daero, Dongdaemun-gu, Seoul, 02447 Korea

**Keywords:** Periodontitis, Transcriptome sequencing, Alternative splicing, Gene expression profile

## Abstract

**Background:**

Periodontitis is the most common chronic inflammatory disease caused by complex interaction between the microbial biofilm and host immune responses. In the present study, high-throughput RNA sequencing was utilized to systemically and precisely identify gene expression profiles and alternative splicing.

**Methods:**

The pooled RNAs of 10 gingival tissues from both healthy and periodontitis patients were analyzed by deep sequencing followed by computational annotation and quantification of mRNA structures.

**Results:**

The differential expression analysis designated 400 up-regulated genes in periodontitis tissues especially in the pathways of defense/immunity protein, receptor, protease, and signaling molecules. The top 10 most up-regulated genes were *CSF3*, *MAFA*, *CR2*, *GLDC*, *SAA1*, *LBP*, *MME*, *MMP3*, *MME-AS1*, and *SAA4*. The 62 down-regulated genes in periodontitis were mainly cytoskeletal and structural proteins. The top 10 most down-regulated genes were *SERPINA12*, *MT4*, *H19*, *KRT2*, *DSC1*, *PSORS1C2*, *KRT27*, *LCE3C*, *AQ5*, and *LCE6A*. The differential alternative splicing analysis revealed unique transcription variants in periodontitis tissues. The EDB exon was predominantly included in *FN1*, while exon 2 was mostly skipped in *BCL2A1*.

**Conclusions:**

These findings using RNA sequencing provide novel insights into the pathogenesis mechanism of periodontitis in terms of gene expression and alternative splicing.

**Electronic supplementary material:**

The online version of this article (doi:10.1186/s40246-016-0084-0) contains supplementary material, which is available to authorized users.

## Introduction

Periodontitis is a chronic inflammatory disease of periodontium, characterized by massive destruction of both soft and hard tissues surrounding the teeth [1]. The current concept for the periodontal diseases involve complex interaction between the microbial biofilm and host immune responses that leads to the alteration of bone and connective tissue homeostasis [2, 3]. Understanding the molecular mechanisms underlying the pathogenesis as well as development of efficient therapeutics is furthermore important since periodontitis is linked to other metabolic and/or systemic diseases including diabetes, cardiovascular diseases, and rheumatoid arthritis [4–6].

The analysis of transcriptome by microarrays has been a valuable tool to study the changes in gene expression profiles in gingival tissues of periodontitis patients [7–9]. However, recent advances in the high-throughput RNA sequencing technology revolutionarily enhanced our understanding on the complexity of eukaryotic transcriptome [10, 11]. RNA sequencing has several key advantages over the hybridization-based microarray techniques. First of all, direct sequencing enables an unbiased approach compared with the microarrays that depends on the predetermined genome sequences. Secondly, RNA sequencing is highly accurate in detecting gene expression with very wide dynamic detection ranges with low background. Thus, RNA sequencing is not only useful to precisely determine gene expression profiles but also particularly powerful to detect novel transcription variants via alternative splicing [10].

In the present study, we analyzed the pooled transcriptome from gingival tissues of periodontitis patients and compared with that of healthy patients. The large sum of novel information on the gene expression profiles as well as novel transcripts through alternative splicing would provide not only insights into the pathogenesis of periodontitis but also basis for the development of biomarkers and therapeutic targets.

## Materials and methods

### Periodontitis patient characteristics and gingival tissue samples

Gingival tissue samples were collected from chronic periodontitis patients or healthy individuals. On the basis of clinical and radiographic criteria, the periodontitis-affected site had a probing depth of ≥4 mm, clinical attachment level of ≥4 mm, and bleeding on probing. A total of 10 gingival samples were collected from 9 periodontal healthy patients who visited Kyungpook National University Hospital. Similarly, a total of 10 periodontitis tissue samples were obtained from 4 periodontitis patients with pocket depth of 4~6 mm and 3 severe periodontitis patients with pocket depth of 7 mm or deeper. The patient characteristics are given in Additional file 1: Table S1. All patients were non-smoking and did not have untreated metabolic/systemic diseases nor associated with infection/autoimmune diseases at the time of tissue collection. The size of 3-mm^2^ gingival biopsies were obtained from the marginal gingiva during periodontal flap surgery and immediately stored in RNAlater solution (Thermo Fisher Scientific, Waltham, MA) at −70 °C after removal of blood by brief washing in phosphate-buffered saline. The study was approved by the institutional review board of the Kyungpook National University Hospital with informed consent from all patients.

### Isolation of RNA and RNA sequencing

Frozen tissues were disrupted in the lysis solution of mirVana RNA isolation kit (Thermo Fisher Scientific) using disposable pestle grinder system (Thermo Fisher Scientific). After RNA extraction, the same amount of total RNA isolated from each individual sample (1 μg) was pooled into 2 groups (healthy and periodontitis) and used for further analysis. The integrity of pooled total RNA was analyzed by Agilent 2100 Bioanalyzer (Agilent Technologies, Santa Clara, CA). After purification of mRNA molecules by poly-T oligo-attached magnetic beads followed by fragmentation, the RNA of approximately 300-bp size was isolated using gel electrophoresis. The cDNA synthesis and library construction was performed using the Illumina Truseq RNA sample preparation kit (Illumina, San Diego, CA), following the manufacturer’s protocol. The PCR-amplified cDNA templates on a flow cell was loaded and sequenced in the HiSeq 2000 sequencing system (Illumina) in the paired-end sequencing mode (2 × 101 bp reads).

### Sequencing data analysis

All sequencing raw reads were aligned to the human genome reference hg19 using the GSNAP alignment tool (2013-11-27) [12]. Only uniquely and properly mapped read pairs were used for further analysis. The differentially expressed genes between gingival tissues from periodontal healthy patients and periodontitis patients were identified using the DESeq R package [13]. Differentially expressed genes were defined as those with changes of at least 2-fold between samples and at a false discovery rate (FDR) cutoff of 5 % based on DESeq adjusted *p* values. The analysis of alternative splicing events was performed using MATS software [14]. The differences in the alternative splicing in genes were considered significant when the inclusion difference between samples was equal or greater than 5 % at a 10 % FDR. Each alternative splicing change of the skipped exon vent was manually inspected in UCSC genome browser using the sequencing data. The functional classification analysis of differentially expressed genes was performed using the PANTHER tools (http://www.pantherdb.org). The GO term and KEGG pathway enrichment analysis was performed as described previously [15]. Briefly, the fraction of genes in a test set associated with each GO category was calculated and compared with that of control set comprised of randomly chosen genes of the same number and length of the test genes. The random sampling was repeated 100,000 times for the calculation of empirical *p* value. The significance of enriched GO terms or KEGG pathways were determined by the *p* value cutoff, which was 1/total number of GO terms considered.

### Validation of differentially expressed genes and alternative splicing events

From the pooled RNA samples, 1 μg of RNA was reversed transcribed using the Superscript II Reverse Transcriptase (Thermo Fisher Scientific). Quantitative real-time PCR analysis was performed by the addition of 1 μg of cDNA and SYBR green master mix in MicroAMP optical tubes using the AB 7500 system (Thermo Fisher Scientific). The expression of genes relative to that of *HPRT1* was determined by the 2^–ΔΔCt^ method [16]. The differential alternative splicing events were confirmed via RT-PCR analysis with the addition of 1 μg of cDNA and Takara premix Taq polymerase (Takara Bio Inc, Shiga, Japan) for 33 cycles of 10 s at 98 °C, 30 s at 60 °C, and 1 min at 72 °C. The primers for the detection of alternative splicing were designed by the PrimerSeq software [17] in order that the PCR product to span the region of exon inclusion/skipping, enabling the differentiation of alternative splicing events by product size. The primer sequences for the real-time RT-PCR analysis of selected genes and those for the RT-PCR detection of alternative splicing events of *FN1* and *BCL2A1* gene were provided in the supplemental tables (Additional file 2: Table S2 and Additional file 3: Table S3).

## Results

### RNA sequencing results

Total RNA was extracted from 10 healthy gingival tissue samples and 10 chronic periodontitis-affected gingival tissues as described above. Then, cDNAs synthesized from the pooled RNA samples of both groups were sequenced using the Illumina HiSeq 2000 system, which generated approximately 80 million pairs of reads of 101 bp in size. When compared with the reference sequence of Genome Reference Consortium GRCh37 (hg19), more than 90 % of read pairs were uniquely mapped on the human genome (Table 1). Gene annotation using the Ensembl (release 75) identified that a total of 36,814 genes have at least 1 read mapped on the exonic regions. Among these, 4800 genes were unique to the periodontitis tissue sample, while 2811 transcripts were detected only in healthy gingival sample.

**Table 1:** Summary of RNA sequencing read mapping results

	Number of total sequencing pairs	Number of unique pairs	Number of unmapped pairs	Percentage of uniquely mapped pairs
Periodontitis tissue	87,118,086	80,778,080	6,340,006	92.72
Healthy tissue	72,014,202	67,035,158	4,979,044	93.09

### Identification and classification of differentially expressed genes between periodontitis and healthy gingiva

**Fig. 1:**
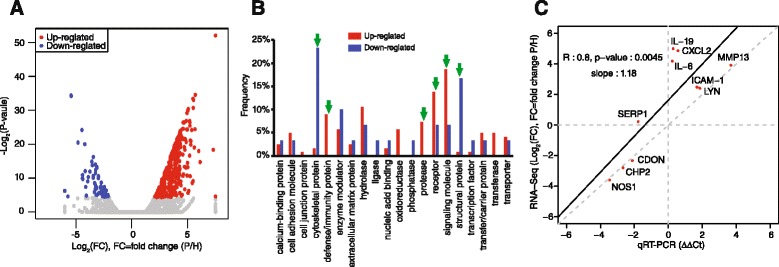
Differential gene expression between periodontitis-affected and healthy gingival tissues. **a** A volcano plot shows the differentially expressed genes. *Red dots* represent the significantly up-regulated genes and *blue dots* stand for the significantly down-regulated genes in periodontitis-affected gingival tissues. The *x*-axis represents the log_2_-transformed gene expression in periodontitis tissues (*P*) divided by that in healthy gingival tissues (*H*). The *y*-axis is the adjusted *p* value (−log_2_) by Benjamini-Hochberg correction. **b** Protein functional classification in differentially expressed genes was performed using the PANTHER tool. The *green arrows* indicate protein functional classes that show significantly different composition (more than 7 % composition difference) between healthy and periodontitis tissues. **c** The expression of selected genes in RNA sequencing data was validated by a real-time RT-PCR analysis. The *x*-axis indicates the −ΔΔCt values and the *y*-axis represents log_2_ (fold changes) obtained by RNA sequencing. The linear regression was performed with Pearson’s correlation coefficient (*R*) and the corresponding *p* value based on the gene expression values by both methods

The differential expression of genes between periodontitis and healthy gingival samples was analyzed by DESeq package [13]. By applying the cutoff of at least twofold change in the number of reads with 5 % FDR, we found a total of 462 genes differentially expressed between the samples (Fig. 1a, volcano plot). While 400 genes were up-regulated in the periodontitis tissue sample, 62 genes were down-regulated compared with the healthy control (Additional file 4: Table S4). Previously, Davanian et al. reported the discovery of 381 genes up-regulated in the periodontitis-affected gingival tissues by RNA sequencing [18]. Notably, 182 genes among them were also found to be up-regulated in the present study (Additional file 5: Figure S1), demonstrating an overlap between the two sets of gene lists when analyzed by a hypergeometric test (*p* < 2.2e^−16^) [19].

The top 20 up-regulated genes listed in Table 2 included cytokines and immune response-related genes (*CSF3*, *CR2*, *LBP*, *CXCL1*, and *IL19*), serum amyloid proteins (*SAA1*, *SAA4*, and *SAA2*), and proteases (*MME*, *MMP3*, *MME-AS1*, and *MMP7*). The 20 most down-regulated genes (Table 3) included peptidase inhibitors (*SERPINA12* and *SPINK9*) and structural proteins (*KRT2*, *KRT27*, *LCE3C*, *LCE6A*, *LCE1B*, *LCE2D*, and *KRT1*).

**Table 2:** Top 20 up-regulated genes in periodontitis tissues

Ensemble ID	Gene symbol	Fold change	*q* value	Description
ENSG00000108342	*CSF3*	181.6	5.9E-21	Colony stimulating factor 3 (granulocyte)
ENSG00000182759	*MAFA*	157.5	8.2E-09	V-MAF avian musculoaponeurotic fibrosarcoma oncogene homolog A
ENSG00000117322	*CR2*	69.6	1.5E-07	Complement component (3D/Epstein Barr virus) receptor 2
ENSG00000178445	*GLDC*	50.8	2.6E-11	Glycine dehydrogenase (decarboxylating)
ENSG00000173432	*SAA1*	46.4	1.8E-14	Serum amyloid A1
ENSG00000129988	*LBP*	45.1	1.4E-05	Lipopolysaccharide binding protein
ENSG00000196549	*MME*	45.0	1.2E-14	Membrane metallo-endopeptidase
ENSG00000149968	*MMP3*	39.6	7.1E-10	Matrix metallopeptidase 3 (stromelysin 1, progelatinase)
ENSG00000240666	*MME-AS1*	38.8	2.1E-09	MME antisense RNA 1
ENSG00000148965	*SAA4*	37.3	4.7E-06	Serum amyloid A4, constitutive
ENSG00000137673	*MMP7*	37.1	4.5E-12	Matrix metallopeptidase 7 (matrilysin, uterine)
ENSG00000130513	*GDF15*	36.6	3.5E-08	Growth differentiation factor 15
ENSG00000134339	*SAA2*	36.4	2.6E-12	Serum amyloid A2
ENSG00000163739	*CXCL1*	33.3	1.6E-13	Chemokine (C-X-C motif) ligand 1 (melanoma growth stimulating activity, alpha)
ENSG00000117215	*PLA2G2D*	32.8	2.1E-04	Phospholipase A2, group IID
ENSG00000142224	*IL19*	32.4	5.3E-07	Interleukin 19
ENSG00000134873	*CLDN10*	31.8	1.8E-07	Claudin 10
ENSG00000255071	*SAA2-SAA4*	31.7	3.0E-11	SAA2-SAA4 readthrough
ENSG00000145113	*MUC4*	31.3	2.6E-11	Mucin 4, cell surface associated

**Table 3:** Top 20 down-regulated genes in periodontitis tissues

Ensemble ID	Gene symbol	Fold change	*q* value	Description
ENSG00000165953	*SERPINA12*	0.015	1.3E-04	Serpin peptidase inhibitor, clade A (alpha-1 antiproteinase, antitrypsin), member 12
ENSG00000102891	*MT4*	0.018	5.5E-04	Metallothionein 4
ENSG00000130600	*H19*	0.023	4.0E-15	H19, imprinted maternally expressed transcript (non-protein coding)
ENSG00000172867	*KRT2*	0.023	5.4E-15	Keratin 2
ENSG00000134765	*DSC1*	0.045	3.6E-11	Desmocollin 1
ENSG00000204538	*PSORS1C2*	0.046	7.2E-06	Psoriasis susceptibility 1 candidate 2
ENSG00000171446	*KRT27*	0.047	9.1E-06	Keratin 27
ENSG00000244057	*LCE3C*	0.053	1.8E-09	Late cornified envelope 3C
ENSG00000161798	*AQP5*	0.055	1.3E-06	Aquaporin 5
ENSG00000235942	*LCE6A*	0.057	5.3E-05	Late cornified envelope 6A
ENSG00000188959	*C9orf152*	0.059	4.2E-04	Chromosome 9 open reading frame 152
ENSG00000196734	*LCE1B*	0.067	5.4E-06	Late cornified envelope 1B
ENSG00000187223	*LCE2D*	0.073	1.5E-06	Late cornified envelope 2D
ENSG00000089250	*NOS1*	0.083	1.4E-07	Nitric oxide synthase 1 (neuronal)
ENSG00000204909	*SPINK9*	0.085	3.1E-07	Serine peptidase inhibitor, Kazal type 9
ENSG00000130595	*TNNT3*	0.091	6.7E-06	Troponin T type 3 (skeletal, fast)
ENSG00000110675	*ELMOD1*	0.091	4.1E-06	ELMO/CED-12 domain containing 1
ENSG00000167768	KRT1	0.091	4.5E-08	Keratin 1
ENSG00000237515	SHISA9	0.097	1.8E-04	Shisa family member 9

To classify the differentially expressed genes into functionally related subgroups, we utilized the PANTHER classification system (http://pantherdb.org). As a result, the 462 differentially expressed genes between periodontitis and healthy gingival tissues were segregated into 20 different classes of proteins. When we compared the composition of these protein classes, there was a significant difference in the number of genes between periodontitis and healthy gingival samples in 6 protein classes. In the periodontitis tissue, genes classified as defense/immunity protein, receptor, protease, and signaling molecules were significantly enriched (Fig. 1b). On the other hand, genes in the categories of cytoskeletal protein and structural protein were predominant in healthy tissue sample compared with periodontitis. Furthermore, functional annotation of GO and KEGG pathway enrichment analyses as previously described [15] revealed enhanced immune responses in the periodontal tissues, including NOD-like receptor signaling, cytokine and chemokine activities, response to lipopolysaccharide, Jak-STAT signaling pathway, and B cell receptor signaling pathway (Additional file 6: Table S5 and Additional file 7: Table S6).

### Validation of differentially expressed genes between periodontitis and healthy gingiva by quantitative real-time PCR analysis

To validate the differential gene expression results by RNA sequencing analysis, we selected 10 up-regulated or down-regulated genes in periodontal tissue and assessed their expression by quantitative real-time RT-PCR analysis. Figure 1c shows that the examination of differential gene expression by both methods is significantly concordant, with the Pearson’s correlation coefficient (*R*) value of 0.81 (*p* = 0.005). Since the current study design employed pooling of samples, we further validated the variations in gene expression in individual samples of healthy and periodontitis patients. The real-time RT-PCR analyses for selected genes (Additional file 8: Figure S2) mostly repeated the RNA sequencing results, showing significant reduction in *NOS1*, *CHP2*, *CDON*, and *MT4*. Similarly, significant elevation was observed in *ICAM1*, *MMP13*, *LYN*, *CSF3*, *MMP3*, *LBP*, and CXCL2 while the expression of IL6 and IL19 only slightly increased. However, a large individual variation was observed in *SERP1* and *KRT2* expression.

### Alternative splicing events in periodontitis and healthy gingival tissues

More than 90 % of human genes are alternatively spliced through different types of splicing [20, 21]. To identify the differential splicing events between the healthy and periodontitis gingival tissues, the inclusion level of alternative spliced exons was compared using the MATS tool [14] based on a statistical model that calculates the difference in the isoform ratio of a gene. The MATS analysis of RNA sequencing data revealed 183 significantly differential alternative splicing events in 155 genes with a cutoff of 5 % inclusion difference and 10 % FDR (Table 4 and Additional file 9: Table S7). The GO and KEGG pathway enrichment analyses for the determination of the biological relevance of those differentially spliced genes showed significant difference in the pathways including RNA splicing regulation, substrate adhesion-dependent cell spreading, response to wound healing, and positive regulation of cell migration (Additional file 10: Table S8 and Additional file 11: Table S9).

**Table 4:** Summary of the differential alternative splicing event analysis

	Alternative 3′ splicing sites	Alternative 5′ splicing sites	Mutual exclusive exon	Retained intron	Skipped exon
Number of total alternative splicing events (genes)	3125 (2177)	2124 (1622)	4424 (2562)	2272 (1800)	32,824 (10,026)
% of total alternative splicing events (45,259)	6.9	4.7	9.8	6.1	72.5
Number of differential alternative splicing events (genes)	10 (10)	4 (4)	34 (32)	82 (77)	53 (42)
% of total differential alternative splicing events (183)	5.5	2.2	18.6	44.8	28.9

**Fig. 2:**
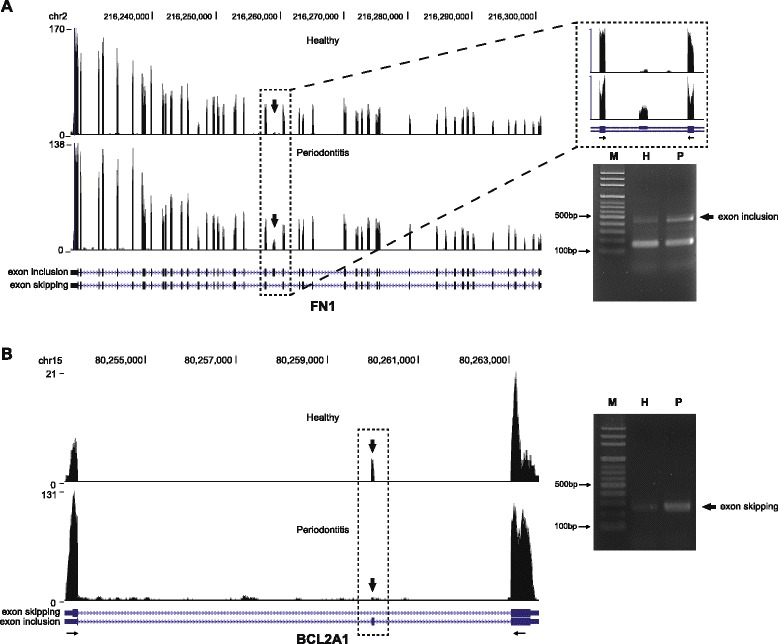
Differential alternative splicing of *FN1* and *BCL2A1*. **a** In the *left panel*, a read distribution plot for *FN1* with differential isoform expression due to the inclusion of EDB domain in periodontitis tissues was shown. The *black boxes* in the annotated isoforms illustrated below the read distributions indicate the exons. *Arrows* indicated the location of EDB exon, which was magnified in the *dotted box* in the upper right panel. In the lower right panel, a reverse transcription-PCR analysis was performed to detect included EDB exon. **b** A read distribution plot for *BCL2A1* with differential isoform expression due to the skipping of exon 2 (*arrows*) in periodontitis tissues was shown in the left panel. In the right panel, a reverse transcription-PCR analysis was performed to detect skipped exon 2. *M* molecular weight marker, *H* healthy gingival tissues, and *P* periodontitis-affected gingival tissues

Among the genes that exhibited prominently novel included exons was *FN1* that encodes one of the major extracellular matrix protein fibronectin [22]. Fibronectin structure consists of 2 nearly identical ~250-kDa glycoprotein subunits with each monomer composed of repetitive units of type I, II, and III domains [23]. The type III domains contain 2 exons called extra domain A (EDA) and extra domain B (EDB), the latter showed significantly increased inclusion in periodontitis gingival tissues compared with healthy samples (Fig. 2a; left panel). The preferential formation of EDB-containing isoform in periodontitis was further corroborated by the RT-PCR analysis designed to amplify the included EDB exon regions (Fig. 2a; right panel). The analysis of alternative splicing events also indicated that *BCL2A1* (BCL2-related protein A1) exhibited prominently skipped exon 2 (Fig. 2b; left panel). RT-PCR analysis designed to amplify the skipped region revealed significantly increased shorter isoform (Fig. 2b; right panel). The individual variation between healthy and periodontitis tissues for these differences in the alternative slicing events was further confirmed by RT-PCR analyses (Additional file 12: Figure S3). For *FN1*, the inclusion of EDB exon was preferentially observed in periodontitis tissues (7/10) compared with healthy tissues (3/8) tested. Similarly, the skipping of exon 2 in *BCL2A1* was predominant in periodontitis tissues (9/10), compared with healthy tissues (2/8).

## Discussion

Recent developments in the RNA sequencing technology and bioinformatics tools enabled elaborate analysis of gene expression in numerous human diseases. However in periodontitis research, most RNA sequencing studies have focused on the identification of microbiome that constitutes periodontal biofilm, with little attention to the host responses against such microbial challenge. The current study provides extensive information on gene expression as well as alternative splicing in periodontitis gingival tissues, which is crucial for the understanding the pathogenesis and development of biomarkers and therapeutic targets. The gene expression analysis revealed 62 down-regulated and 400 up-regulated genes in periodontitis tissues, suggesting the effectiveness of mRNA sequencing as a tool to scrutinize the differential gene expression during the development of periodontitis. Davanian et al. previously reported a series of up-regulated genes as well as enriched biological pathways in periodontitis [18]. When we compared these results with ours, the current results only partially overlap in terms of differential gene expression, possibly originated from the difference in the ethnic group of the subjects as well as in the methods to eliminate individual fluctuations in the gene expression. For example, Davanian et al. used healthy gingival tissue of the same periodontitis-affected individual as healthy control tissue. However, in the current study, the healthy and periodontitis tissues were pooled, allowing the dilution of individual differences in the gene expression. Indeed, the RNA sequencing analysis of pooled samples proved effective, since the expression levels of genes (except *IL6* and *IL19*) identified as differentially expressed by RNA sequencing were also significantly different between healthy and periodontitis samples, when we confirmed by real-time PCR analysis of individual samples (Additional file 8: Figure S2). Most of the top 20 up-regulated genes in periodontitis tissue (Table 2) were associated with inflammation and tissue degradation. Notably, serum amyloid A isoforms consisted 3 of 20 most up-regulated mRNAs, supporting the notion that these can serve as biomarkers for periodontitis-associated acute as well as chronic inflammation [24].

Until recently, gene expression analyses mostly focused on the genes whose expression was significantly increased in periodontitis. In line with this, 18 of top 20 up-regulated genes were associated with periodontal disease at least once by previous studies. The current study revealed 2 novel genes highly overexpressed in periodontitis tissues compared with healthy control. *MAFA* is a subgroup member of the basic leucine-zipper family transcription factor prominently known for its role in glucose-responsive insulin secretion [25]. *CLDN10* is an ion channel-forming member of claudin family, which is a constituent of tight junction [26]. The role of these genes in periodontitis is of great interest and requires further investigation.

In contrast to the highly expressed genes in periodontitis, fewer highlights have been drawn on the genes down-regulated in periodontal diseases. In accordance, most of the top 20 down-regulated genes (Table 3) have not been studied with regard to periodontitis, although investigating the role of those genes in periodontitis compared with that in normal tissues would greatly enhance our knowledge regarding the pathogenesis of periodontal diseases. Notably, keratin (*KRT2*, *KRT27*, and *KRT1*) and late cornified envelope (*LCE3C*, *LCE6A*, *LCE1B*, and *LCE2D*) genes constituted significant part of the down-regulated genes, suggesting the loss of epithelial barrier [27]. The causal relationship between the loss of these genes and the development of periodontal diseases requires further investigation.

It has long been suggested that different sites in the same individual exhibit different patterns of disease progression, morphology, and often response to therapy [28]. In addition, the oral microbiota responsible for the induction of periodontal diseases is distinct from site-to-site in the same individual [29, 30]. Accordingly, it is recommended to design clinical studies based on individual sites rather than individual person [31]. In agreement of this notion, the analysis of gene expression in individual sites by real-time RT-PCR (Additional file 8: Figure S2) revealed site-specific variation. In different sites from the same periodontitis patients (P2: P3, P7: P8, and P9: P10), it was clearly noticeable that *MMP3*, *MMP13*, and *LBP* expressions differ in a site-specific manner. An individual RNA sequencing study with larger number of patients is ongoing, which will further provide detailed information on the site specificity of periodontitis.

The gene ontology and KEGG pathway enrichment analyses revealed both innate and adaptive immune responses in the periodontal tissues, including NOD-like receptor signaling, response to lipopolysaccharide, cytokine and chemokine activities, and B cell receptor signaling pathways (Additional file 6: Table S5 and Additional file 7: Table S6). The NOD1 and NOD2 have been suggested to mediate the sensing of periodontal bacteria [32]. In addition, NOD2 has been linked to the *P. gingivalis*-induced bone resorption, since *NOD2* knockout mice were protected from bone loss in a periodontitis model [33]. Bellibasakis and Johansson showed that a periodontal pathogen *A. actinomyceptemcomitans* regulated NLRP3 and NLRP6 expression in human mononuclear cells [34]. Considering the existence of 22 human NOD-like receptor protein members and their crucial functions in immune diseases, it will be of great interest and importance to elucidate the involvement of these receptors in the pathogenesis of periodontitis.

In the periodontitis lesions, it has been estimated that more than 75 % of infiltrating immune cells are plasma cells and B cells, suggesting the importance of these cells in adaptive immunity during the development of periodontitis [35]. In accordance, molecules involved in B cell activation including *CD79*, *CD19*, *Lyn*, and *CR2* were significantly increased in periodontitis tissue. An increasing body of evidence indicates that B cells with autoreactive propensities might be linked to tissue destruction in periodontitis [36, 37]. Indeed, recent reports demonstrated that B cell-deficient mice were protected from alveolar bone loss in experimental periodontitis [38, 39].

Numerous studies attempted to delineate the role of T helper (Th) cell subsets in human periodontitis by examining the cytokine mRNA levels by RT-PCR, flow cytometry, and immunohistochemistry. However, those studies are incoherent in terms of Th1 and Th2 cytokine expression, although the Th17 cytokines are consistently increased [37]. The current study revealed that the levels of Th1 cytokines *IFNG* and *IL12* did not change between healthy and periodontitis-affected gingival samples while that of *TNF* slightly increased in periodontitis (Additional file 13: Table S10). The Th2 cytokines *IL10* and *IL33* remained unaltered in periodontitis patients. Interestingly, Th17 cytokines *IL6*, *IL23A*, and *IL17C* significantly increased in gingival tissues from periodontitis patients compared with those of healthy control, supporting the concept of Th17 cells as crucial mediators of inflammation, although it is still controversial whether these cells contribute to tissue destruction or protection in periodontitis [40, 41].

Alternative splicing of genes contribute to the diversity of proteome as well as genome evolution, control of developmental processes, and physiological regulation of various biological systems [42]. Not surprisingly, dysregulation of alternative splicing is often linked to various human diseases such as cancer, metabolic, neurological, and skeletal diseases [43–47]. However, alternative splicing events in the context of periodontitis has rarely been investigated. The current study uncovered significant differential alternative splicing events in *BCL2A1* and *FN1. BCL2A1* is a target gene of NF-kB, implicated in the survival of leukocytes thereby inflammation [48]. However, the role of alternative splicing on the activity of the protein has not been suggested until the present. Interestingly, recent discovery showed that *BCL2A1* was increased not only in periodontitis but also in systemic diseases such as cardiovascular diseases and ulcerative colitis [49]. Therefore, research regarding the multiple layers of regulatory mechanisms including mRNA expression and alternative splicing of *BCL2A1* are required to fully understand the role of this gene during the pathogenesis of periodontitis.

Parkar et al. previously suggested that *FN1* is differentially spliced in periodontitis [50]. Interestingly, the authors reported exon skipping of both EDA and EDB domain in periodontitis, while the current study showed conspicuously increased inclusion of EDB domain. Although whether these differences originated from the use of periodontal ligament [51] versus gingival tissues (the present study) yet to be cleared, it would be of great interest to fully identify the role of fibronectin isoforms in the pathogenesis of periodontitis considering the suggested role of EDA- and EDB-containing isoforms of fibronectin during embryonic development and tissue repair [23, 51].

In conclusion, the current study presented novel gene expression profiles as well as alternative splicing in gingival tissues from periodontitis patients by RNA sequencing experiments. Considering its effectiveness for whole transcriptome analysis, the use of RNA sequencing in periodontitis research would facilitate the elucidation of pathogenesis.

## Abbreviations

EDA, extra domain A; EDB, extra domain B; FDR, false discovery rate; GO, gene ontology; KEGG, kyoto encyclopedia of genes and genomes; PANTHER, protein analysis through evolutionary relationships

## Additional files

Additional file 1: Table S1. The characteristics of patients involved in the current study. The information on age, gender, and disease severity is given in this table. (DOCX 54 kb) Additional file 2: Table S2. Primer sequences used for the real-time RT-PCR validation of RNA sequencing differential gene expression results. The primer sequences are given in this table. (DOCX 89 kb) Additional file 3: Table S3. Primer sequences used for the RT-PCR validation of RNA sequencing alternative splicing results. The primer sequences are given in this table. (DOCX 50 kb) Additional file 4: Table S4. The full list of differentially expressed genes in healthy and periodontitis tissues. This excel file contains the full list of deferentially expressed genes. (XLSX 43 kb) Additional file 5: Figure S1. Comparison of up-regulated genes in periodontitis with those of the previous study by Davanian et al. The Venn diagram shows the number of genes unique for each study and that of commonly detected genes. (PDF 388 kb) Additional file 6: Table S5. The GO term analysis of genes of up- and down-regulated genes in periodontitis. This excel file contains the list of the differentially expressed genes, categorized according to the GO terms. (XLSX 21 kb) Additional file 7: Table S6. The KEGG pathway analysis of genes of up- and down-regulated genes in periodontitis. This excel file contains the list of the differentially expressed genes, categorized according to the KEGG pathway enrichment analysis. (XLSX 11 kb) Additional file 8: Figure S2. The expression levels of selected genes in individual samples. The individual variation in gene expression was examined by real-time RT-PCR analysis of individual healthy and periodontitis samples. The *p* values of the Wilcoxon rank-sum test between healthy and periodontitis groups are given in each graph. (PDF 566 kb) Additional file 9: Table S7. The full list of differential alternative splicing events in healthy and periodontitis tissues. This excel file contains the full list of exons which were included or excluded in periodontitis. (XLSX 32 kb) Additional file 10: Table S8. The GO term analysis of genes with differential alternative splicing in periodontitis. This excel file contains the list of the genes with differential alternative splicing, categorized according to the GO terms. (XLSX 32 kb) Additional file 11: Table S9. The KEGG pathway analysis of genes with differential alternative splicing in periodontitis. This excel file contains the list of the genes with differential alternative splicing, categorized according to the KEGG pathway enrichment analysis. (XLSX 10 kb) Additional file 12: Figure S3. The alternative splicing events in individual samples. The individual variation in alternative splicing events in *FN1* and *BCL2A1* was examined by RT-PCR analysis of individual healthy and periodontitis samples. (PDF 419 kb) Additional file 13: Table S10. The expression of Th1, Th2, and Th17 cytokines in healthy and periodontitis tissues. This excel file contains the selected list of the Th1, Th2, and Th17 cytokine genes with their expression levels in healthy and periodontitis tissues. (XLSX 37 kb)
