# Supplementary material for: Transcriptome sequencing of gingival biopsies from chronic periodontitis patients reveals novel gene expression and splicing patterns
Source: Hum Genomics. 2016 Aug 17;10:28. doi: 10.1186/s40246-016-0084-0 (PMC4988046; doi:10.1186/s40246-016-0084-0)
Supplement: Additional file 1: Table S1. — The characteristics of patients involved in the current study. The information on age, gender, and disease severity is given in this table. (DOCX 54 kb) [file 40246_2016_84_MOESM1_ESM.docx]

**Table S1.** The characteristics of patients included in the current study.

| **Healthy/Periodontitis** | **Gender** | **Age** | **Pocket depth (mm)** | **Sample #** |
| --- | --- | --- | --- | --- |
| Healthy | M | 43 | ≦3 | H1 |
| Healthy | M | 45 | ≦3 | H9^*^ |
| Healthy | M | 48 | ≦3 | H2 |
| Healthy | M | 51 | ≦3 | H3 |
| Healthy | M | 60 | ≦3 | H4 |
| Healthy | F | 21 | ≦3 | H5 |
| Healthy | F | 39 | ≦3 | H6 |
| Healthy | F | 41 | ≦3 | H7 |
| Healthy | F | 43 | ≦3 | H8, H10^*^ |
| Moderate Periodontitis | M | 29 | 4~6 | P1 |
| Moderate Periodontitis | F | 42 | 4~6 | P2, P3 |
| Moderate Periodontitis | F | 48 | 4~6 | P4 |
| Moderate Periodontitis | F | 63 | 4~6 | P5 |
| Severe Periodontitis | M | 41 | ≧7 | P6 |
| Severe Periodontitis | M | 61 | ≧7 | P7, P8 |
| Severe Periodontitis | F | 50 | ≧7 | P9, P10 |

*, These healthy tissue samples were excluded in the individual analysis of gene expression by real-time RT-PCR (Fig. S2) and alternative splicing events by RT-PCR (Fig. S3) due to the insufficient amount of RNA for subsequent experiments after RNA sequening.
