# Supplementary material for: Transcriptome sequencing of gingival biopsies from chronic periodontitis patients reveals novel gene expression and splicing patterns
Source: Hum Genomics. 2016 Aug 17;10:28. doi: 10.1186/s40246-016-0084-0 (PMC4988046; doi:10.1186/s40246-016-0084-0)
Supplement: Additional file 2: Table S2. — Primer sequences used for the real-time RT-PCR validation of RNA sequencing differential gene expression results. The primer sequences are given in this table. (DOCX 89 kb) [file 40246_2016_84_MOESM2_ESM.docx]

**Table S1.** Primer sequences used in the real-time RT-PCR validation of RNA sequencing deferential expression analysis.

| **Gene name** | **Genebank accession #** | **Primer sequence** |
| --- | --- | --- |
| *NOS1* | NM_000620 | F: 5'-AGTGTCCACGCTTCCTCAAG-3'  R: 5'-GTCTTCAGGCCTCCTTGCAT-3' |
| *CHP2* | NM_022097 | F: 5'-TCTTGGCTCATTTTCGCCCT-3'  R: 5'-GCCTGGAGATCTTCCCATCG-3' |
| *CDON* | NM_001243597 | F: 5'-GAGCTGTGTATGGGAAGGAGAC-3'  R: 5'-CTGTCGGGCTGTCTAAAGGA-3' |
| *SERP1* | NM_003012 | F: 5'-TGGCCCGAGATGCTTAAGTG-3'  R: 5'-CTCGTTGTCACAGGGAGGAC-3' |
| *CXCL2* | NM_002089 | F: 5'-TGTGAAGGTGAAGTCCCCCG-3'  R: 5'-CTTAACCATGGGCGATGCG-3' |
| *IL6* | NM_000600 | F: 5'-TGCAATAACCACCCCTGACC-3'  R: 5'-ATTTGCCGAAGAGCCCTCAG-3' |
| *ICAM1* | NM_000201 | F: 5'-CCCACAGTCACCTATGGCAA-3'  R: 5'-GAGACCTCTGGCTTCGTCAG-3' |
| *MMP13* | NM_002427 | F: 5'-AGGAGCATGGCGACTTCTAC-3'  R: 5'-AGACCTAAGGAGTGGCCGAA-3' |
| *IL19* | NM_153758 | F: 5'-CTGTTCCACGGGGCATGAAG-3'  R: 5'-TGGGAAGGTGTCCTTAGCTTG-3' |
| *LYN* | NM_002350 | F: 5'-CCCGGACGACTTGTCTTTCA-3'  R: 5'-CAAAAGCTGCCTTTCTGCGT-3' |
