# Supplementary material for: Transcriptome sequencing of gingival biopsies from chronic periodontitis patients reveals novel gene expression and splicing patterns
Source: Hum Genomics. 2016 Aug 17;10:28. doi: 10.1186/s40246-016-0084-0 (PMC4988046; doi:10.1186/s40246-016-0084-0)
Supplement: Additional file 3: Table S3. — Primer sequences used for the RT-PCR validation of RNA sequencing alternative splicing results. The primer sequences are given in this table. (DOCX 50 kb) [file 40246_2016_84_MOESM3_ESM.docx]

**Table S2.** Primer sequences used in the RT-PCR validation of RNA sequencing differential alternative splicing analysis.

| **Ensemble ID** | **Gene symbol** | **Target coordinates** | **Primer sequence** | **Product size** |
| --- | --- | --- | --- | --- |
| ENSG00000115414 | *FN1* | chr2:216257653-216257926 | F: 5'-ATGCTGATCAGAGCTCCTGC-3'  R: 5'-TGTTGGTGAATCGCAGGTCA-3' | 429 bp (inclusion)  156 bp (skipping) |
| ENSG00000140379 | *BCL2A1* | chr15:80259978-80260034 | F: 5'-AAATTGCCCCGGATGTGGAT-3'  R: 5'-AGGCCGGTTTCACAATATGGA-3' | 299 bp (inclusion)  243 bp (skipping) |
