# Supplementary figures and images for: Transcriptome sequencing of gingival biopsies from chronic periodontitis patients reveals novel gene expression and splicing patterns
Source: Hum Genomics. 2016 Aug 17;10:28. doi: 10.1186/s40246-016-0084-0 (PMC4988046; doi:10.1186/s40246-016-0084-0)

Figure S1

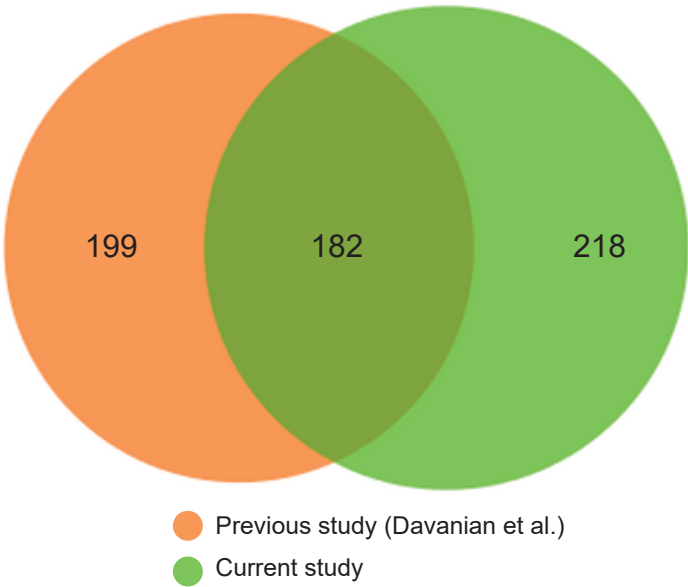

Supplement: Additional file 5: Figure S1. — Comparison of up-regulated genes in periodontitis with those of the previous study by Davanian et al. The Venn diagram shows the number of genes unique for each study and that of commonly detected genes. (PDF 388 kb) [file 40246_2016_84_MOESM5_ESM.pdf]

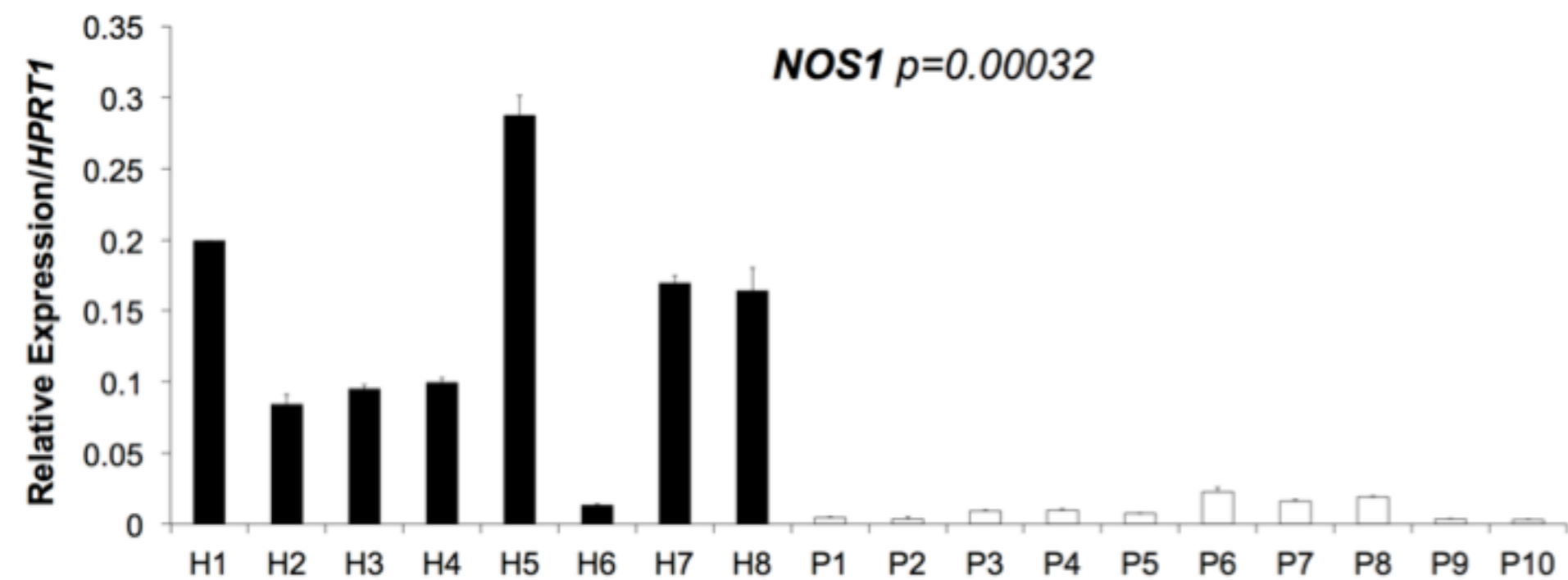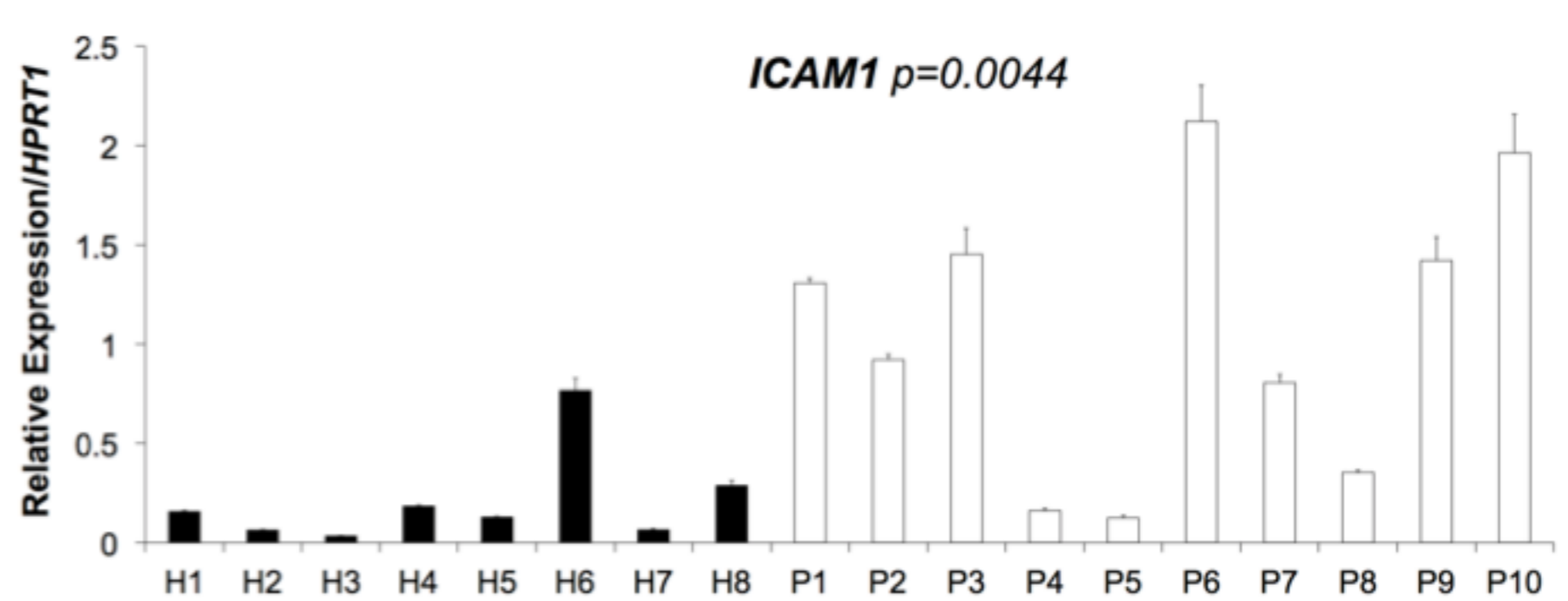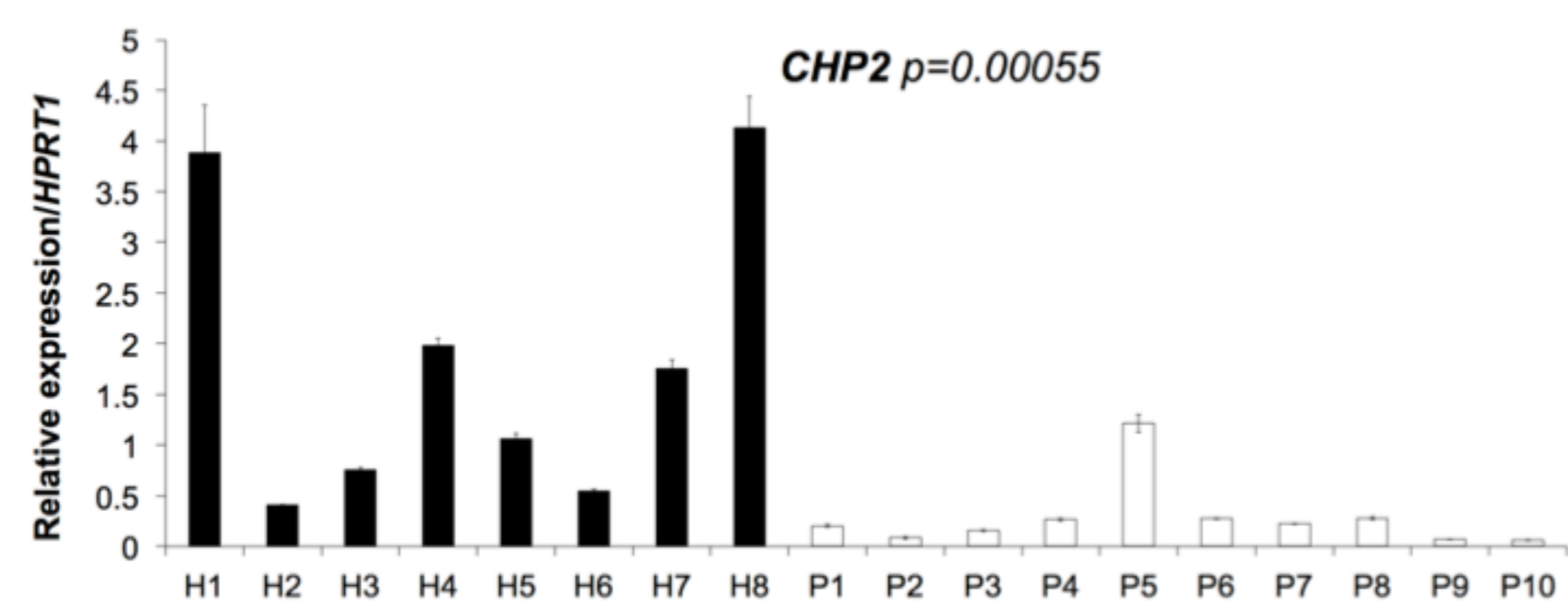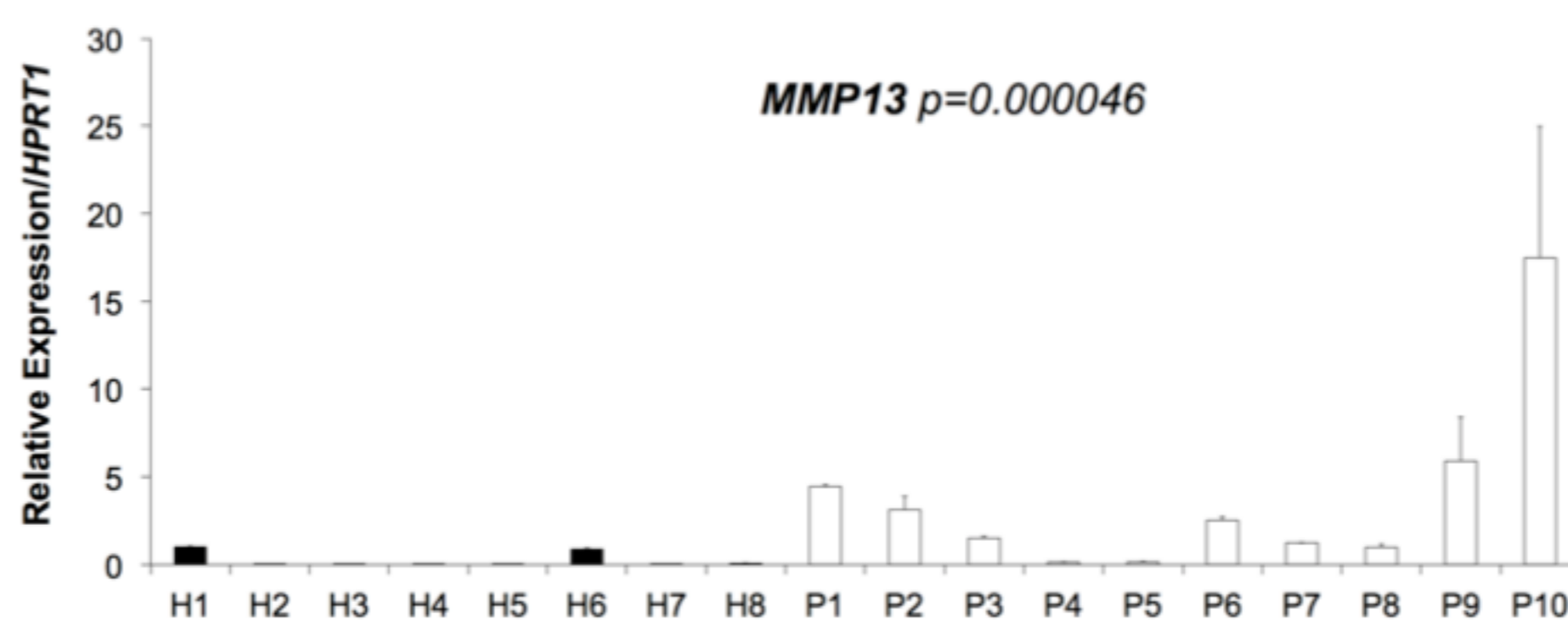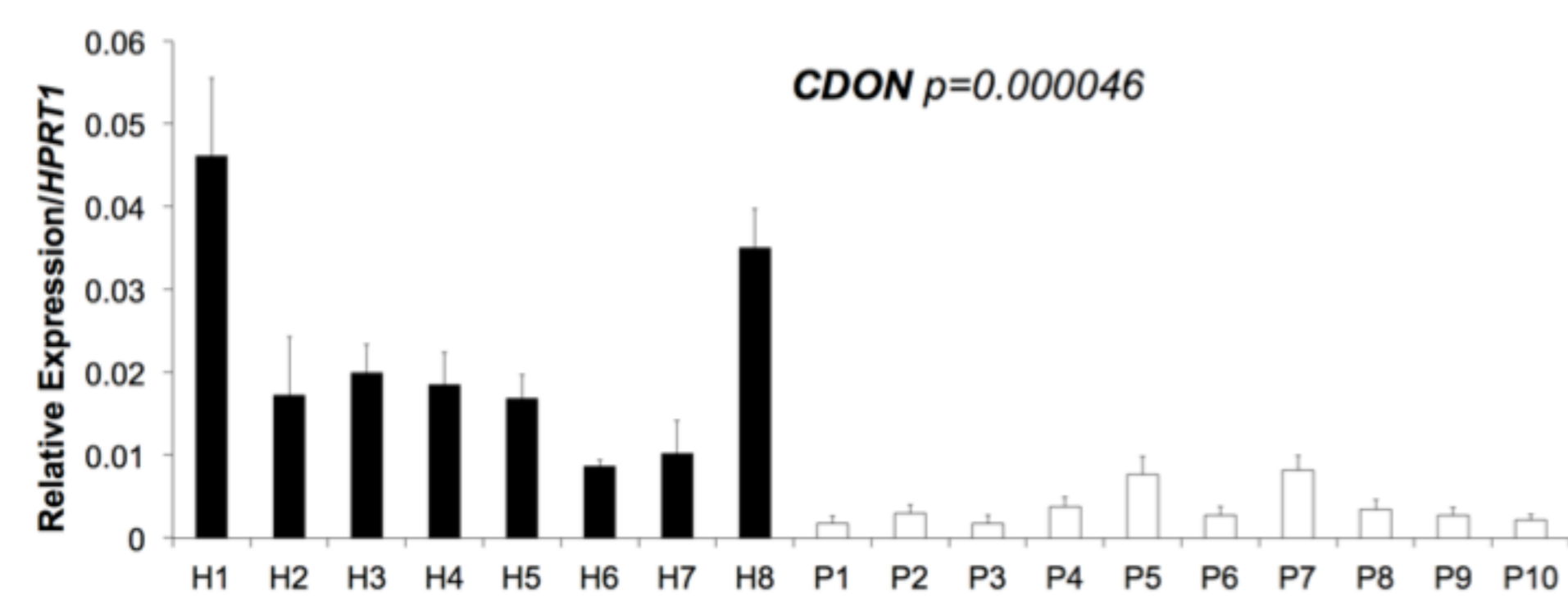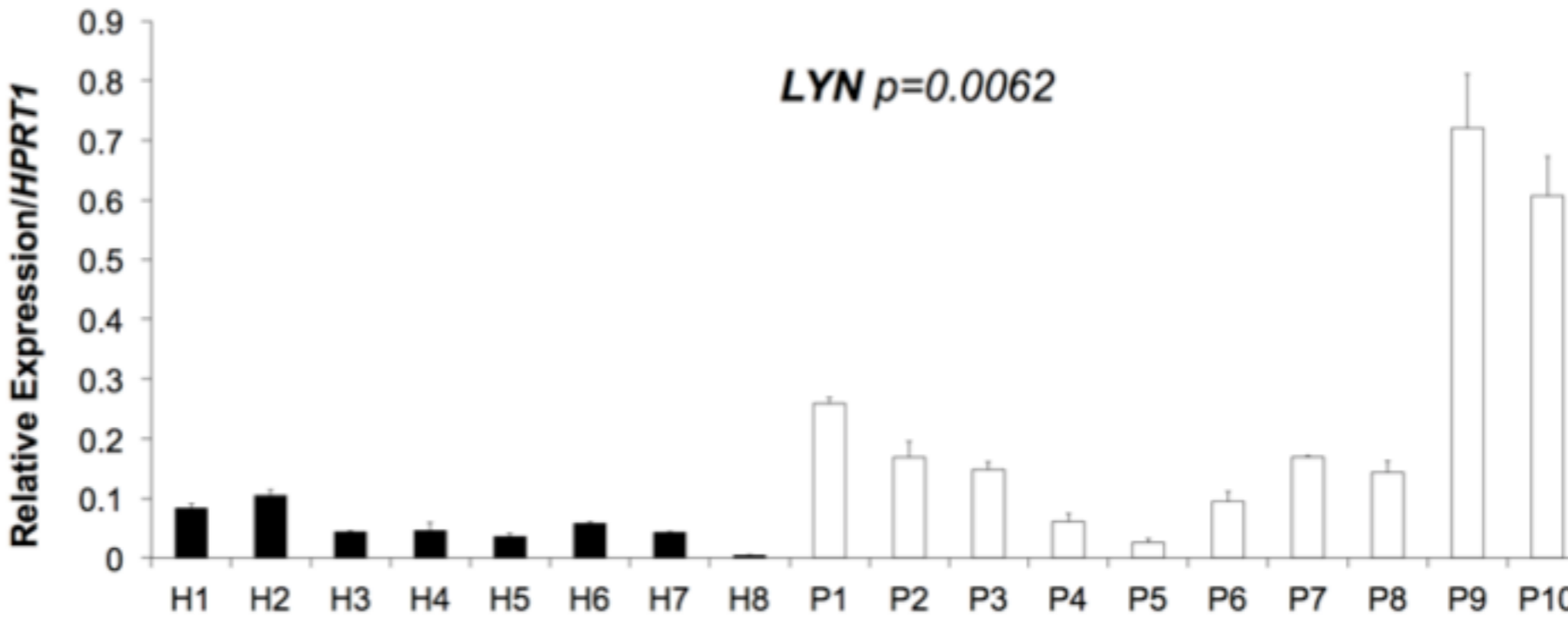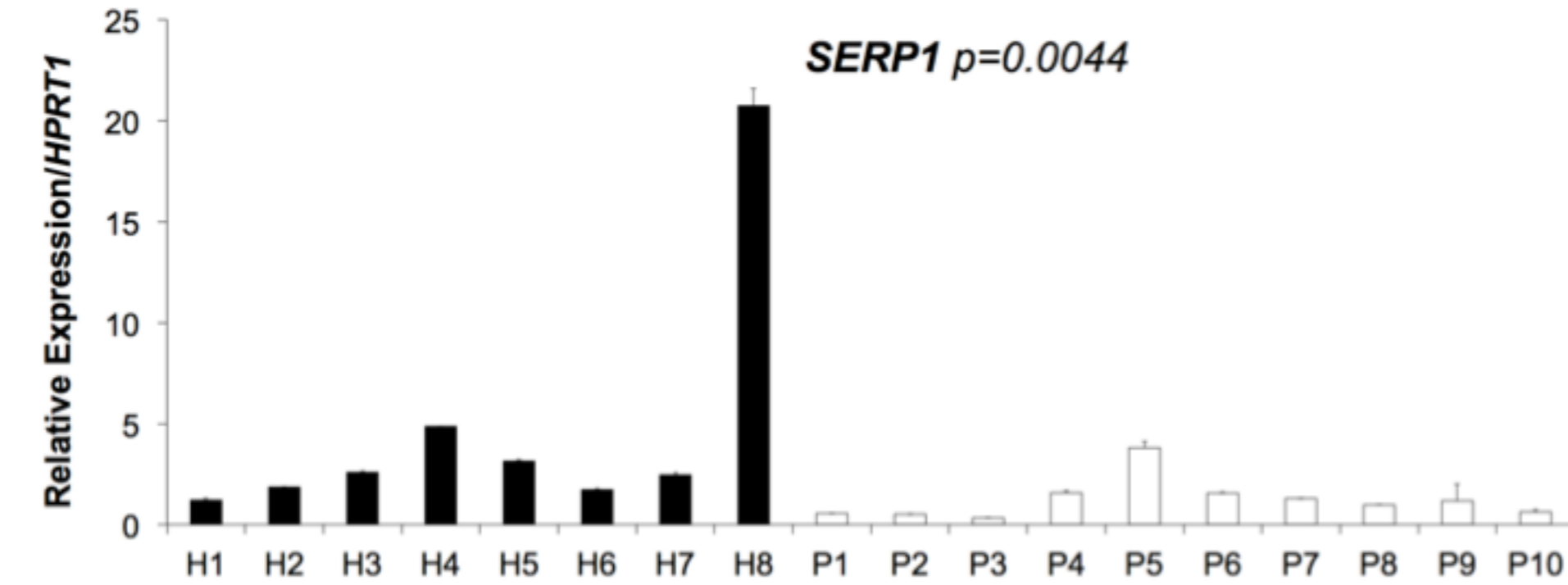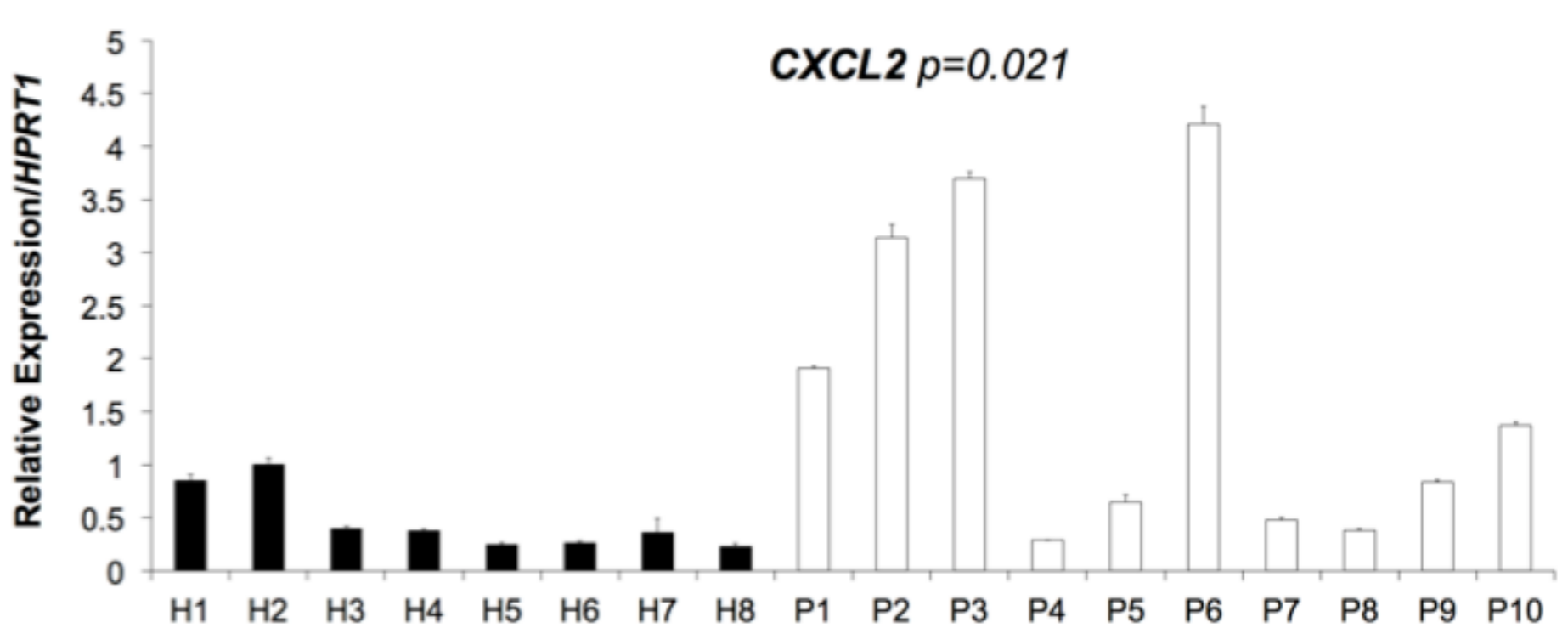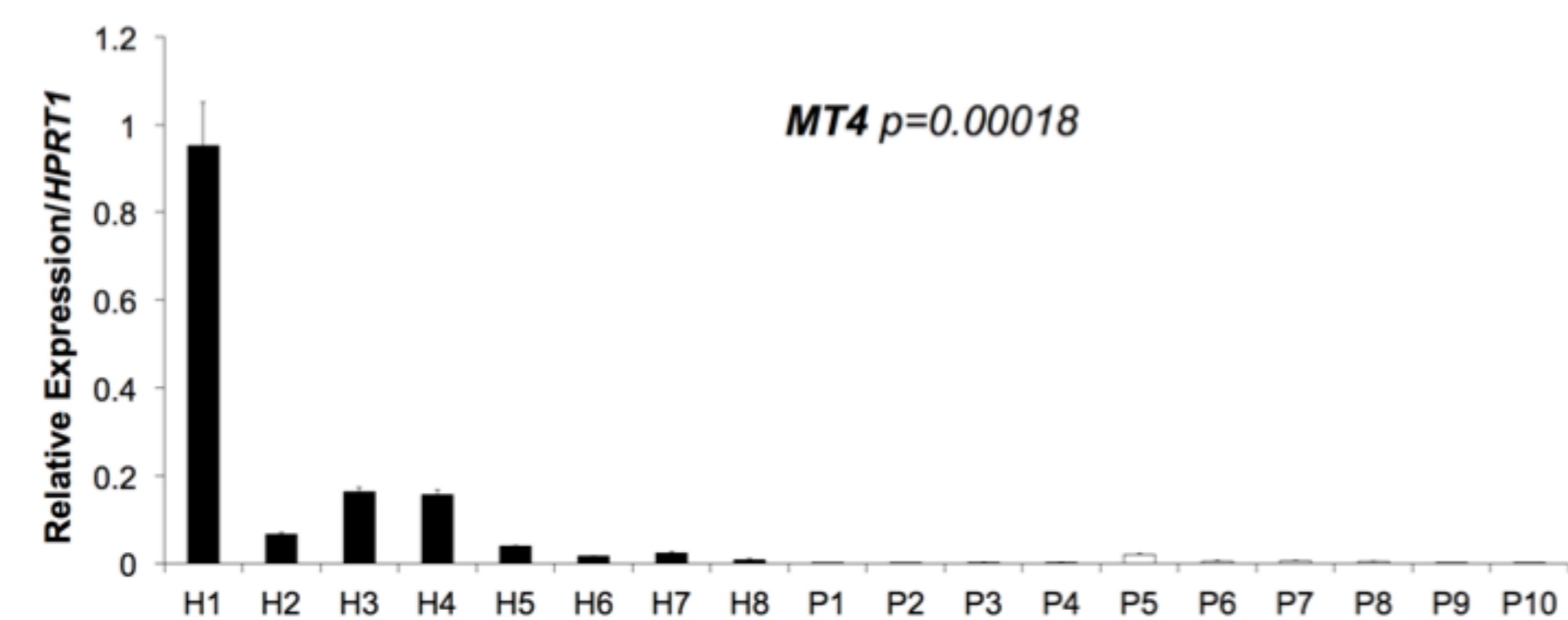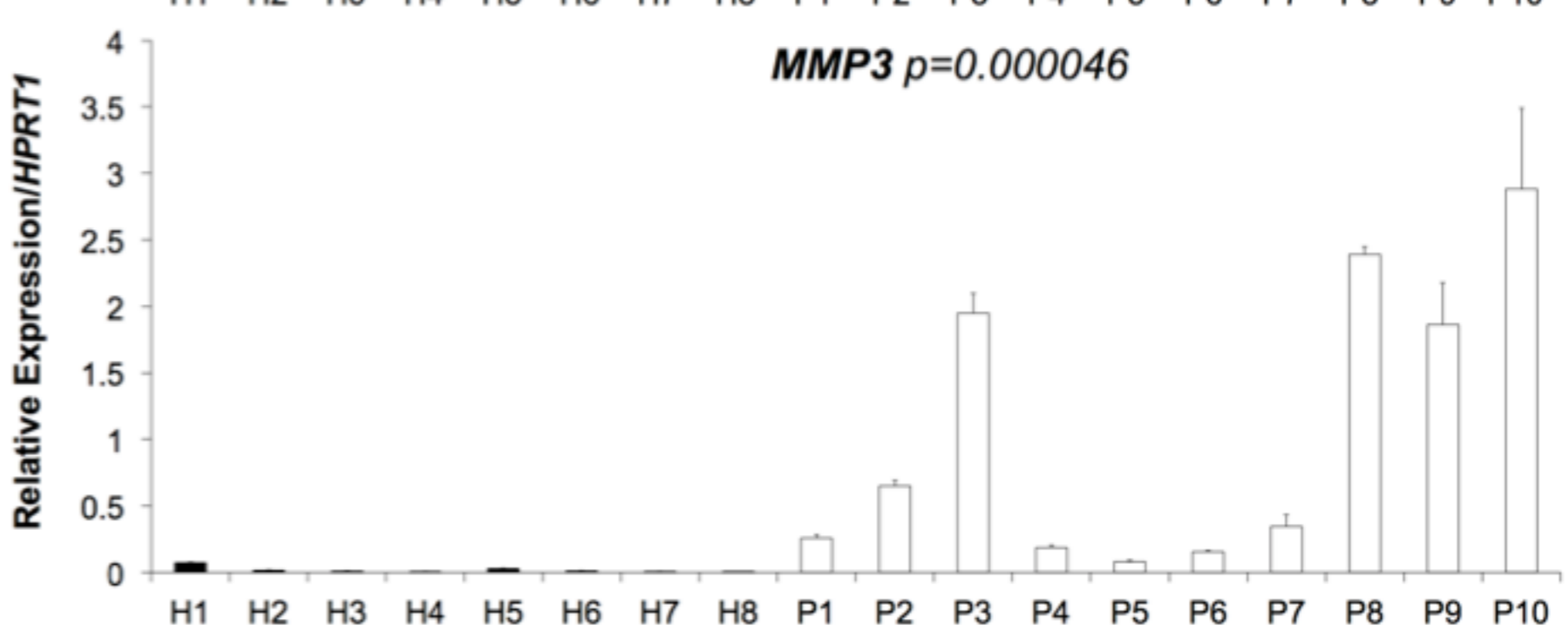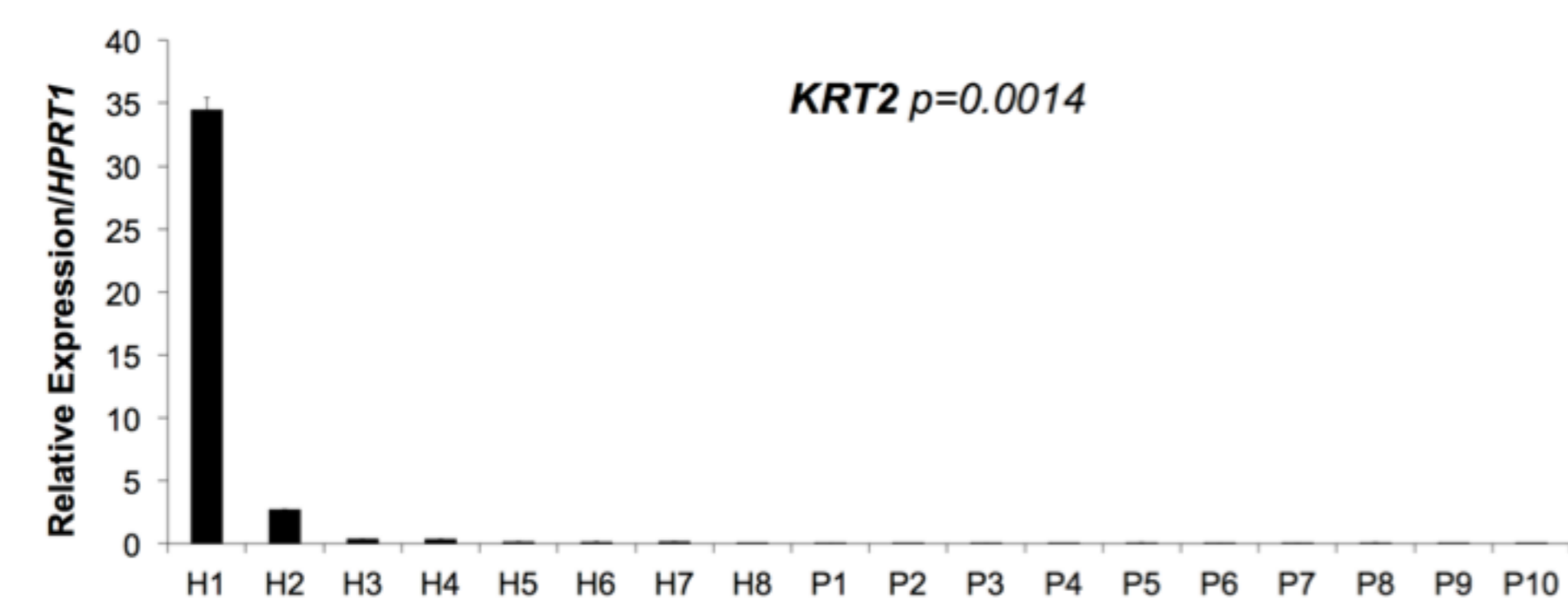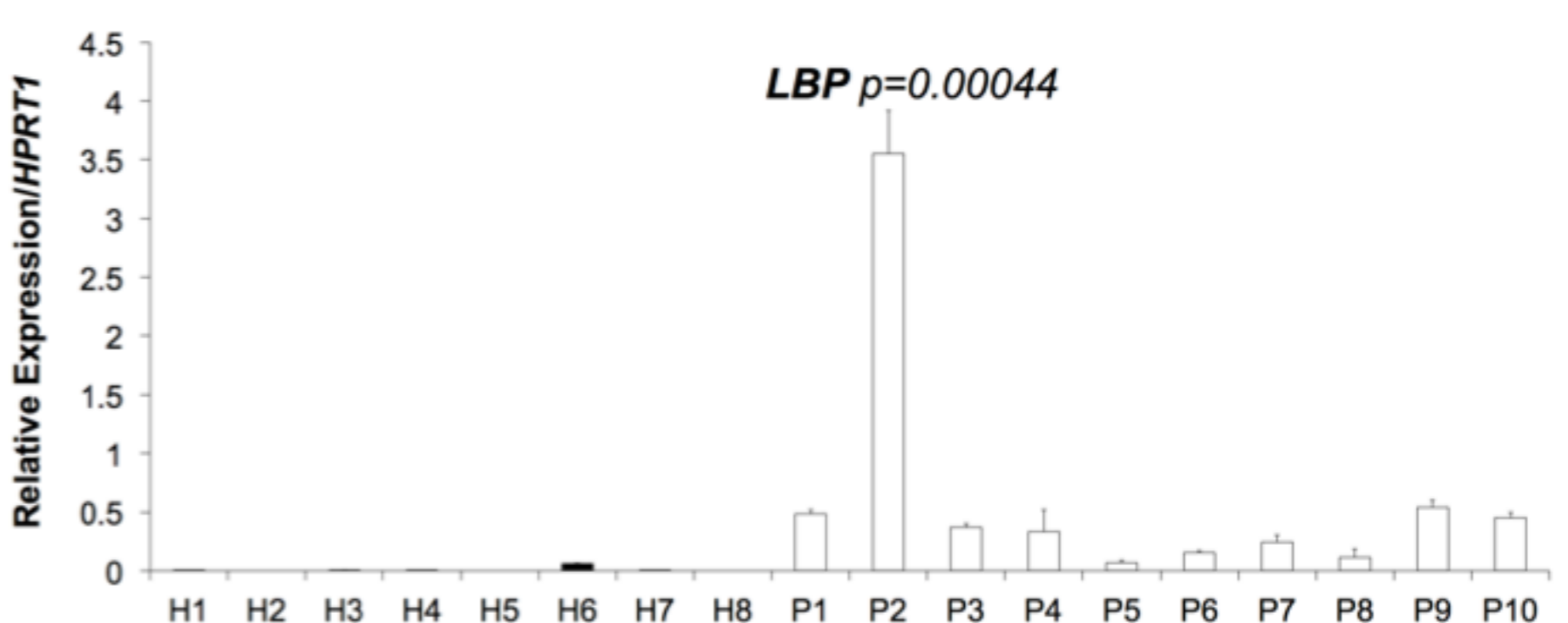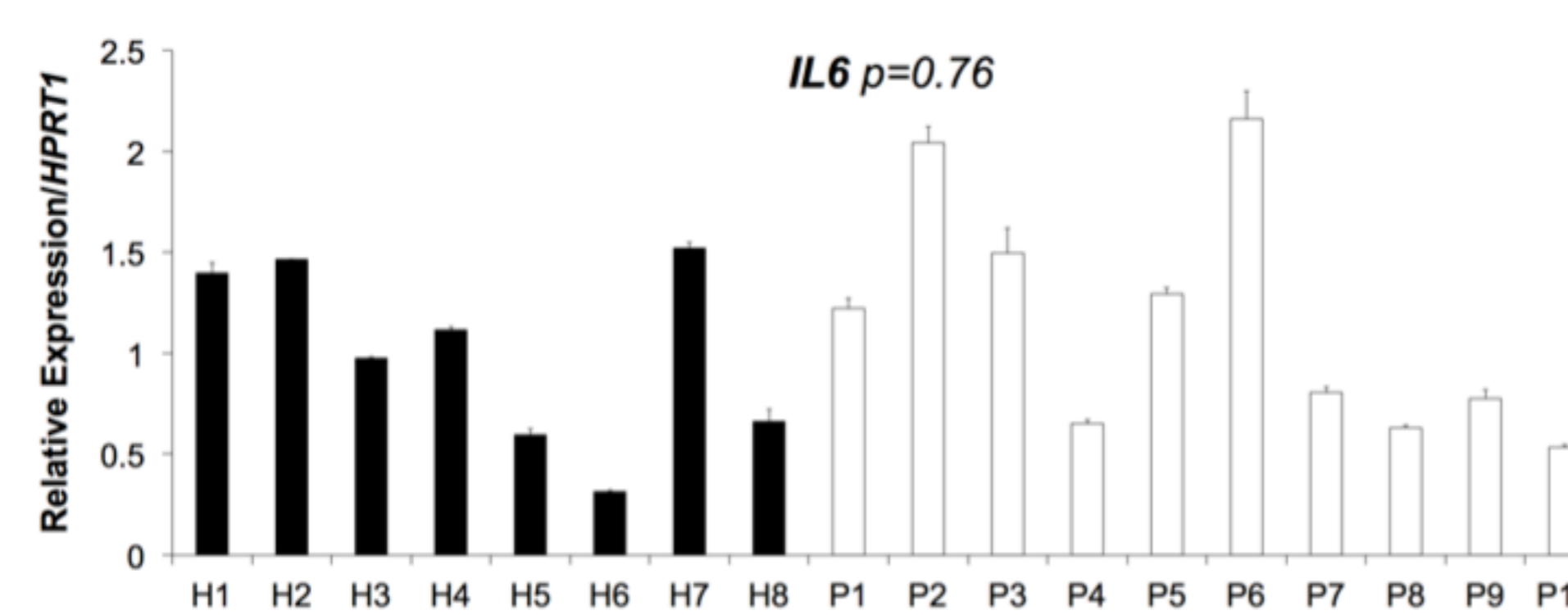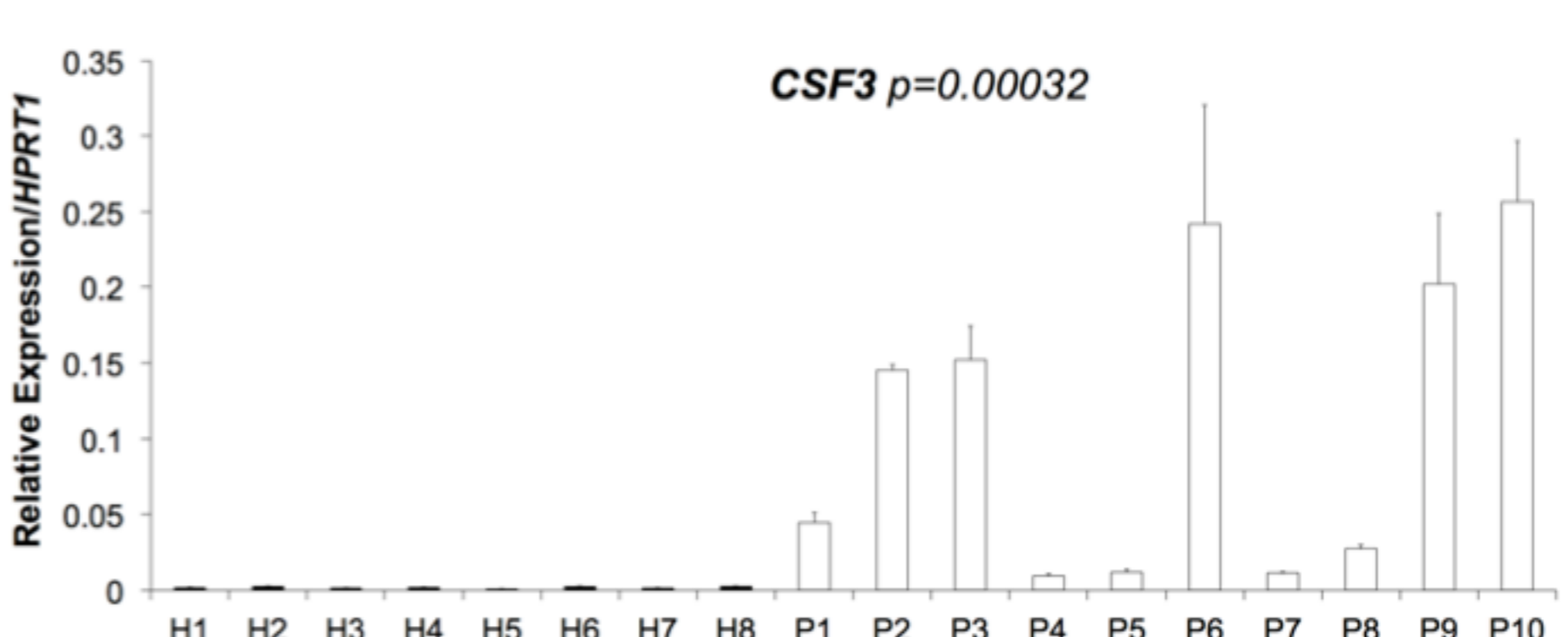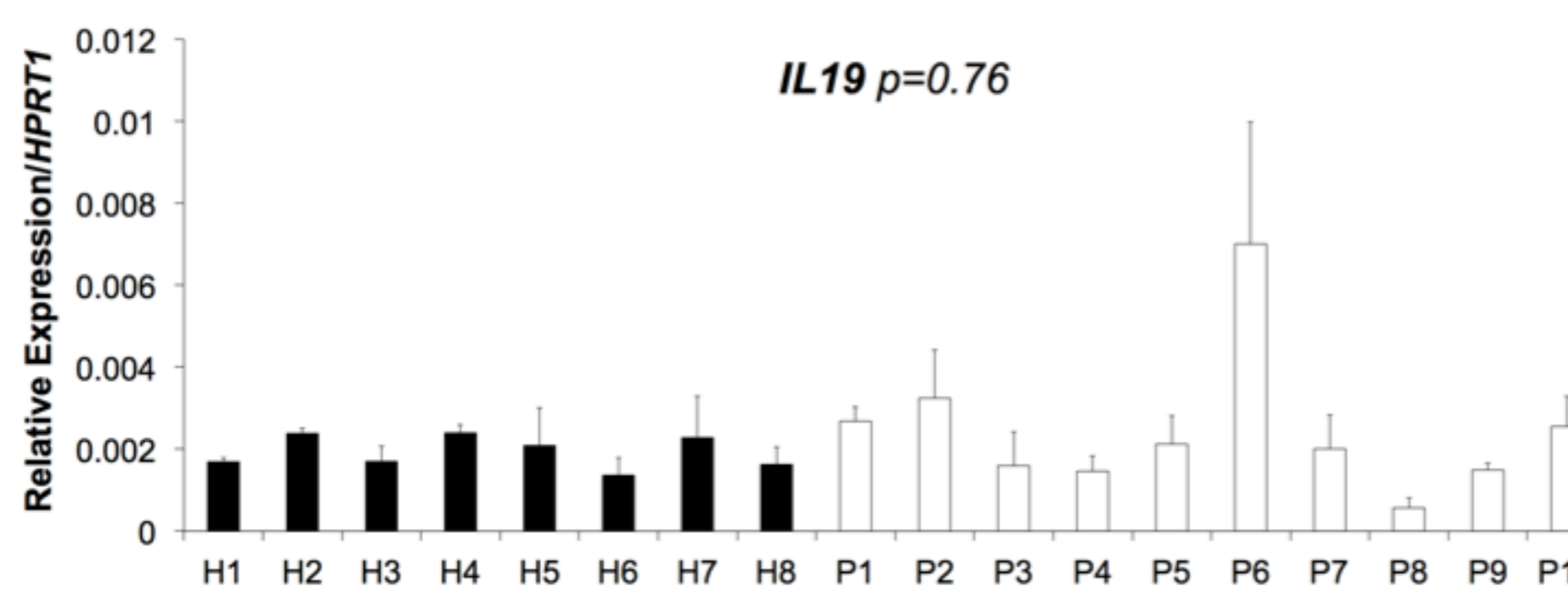

Supplement: Additional file 8: Figure S2. — The expression levels of selected genes in individual samples. The individual variation in gene expression was examined by real-time RT-PCR analysis of individual healthy and periodontitis samples. The p values of the Wilcoxon rank-sum test between healthy and periodontitis groups are given in each graph. (PDF 566 kb) [file 40246_2016_84_MOESM8_ESM.pdf]

*FN1*

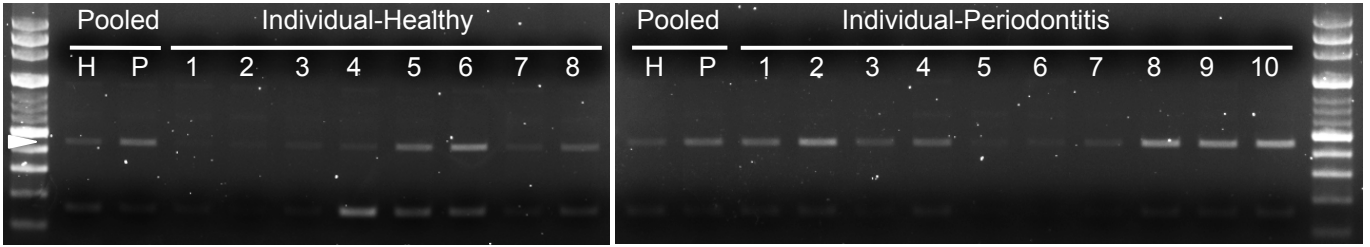

***BCL2A1***

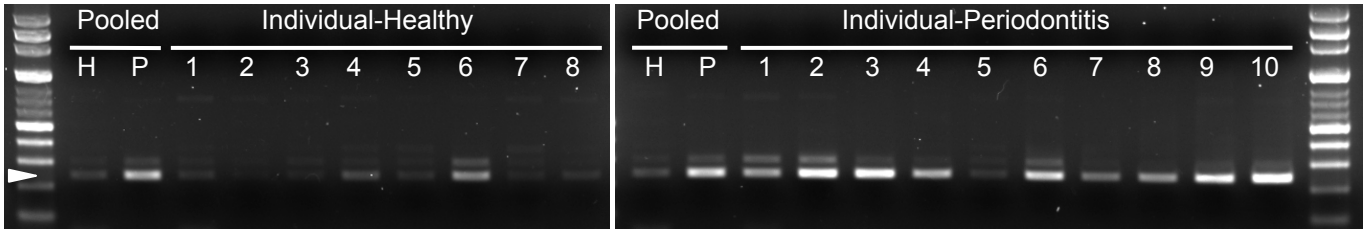

Supplement: Additional file 12: Figure S3. — The alternative splicing events in individual samples. The individual variation in alternative splicing events in FN1 and BCL2A1 was examined by RT-PCR analysis of individual healthy and periodontitis samples. (PDF 419 kb) [file 40246_2016_84_MOESM12_ESM.pdf]
